# GLP1-GIP receptor co-agonists: a promising evolution in the treatment of type 2 diabetes

**DOI:** 10.1007/s00592-024-02300-6

**Published:** 2024-06-03

**Authors:** Stefano Ciardullo, Mario Luca Morieri, Giuseppe Daniele, Teresa Vanessa Fiorentino, Teresa Mezza, Domenico Tricò, Agostino Consoli, Stefano Del Prato, Francesco Giorgino, Salvatore Piro, Anna Solini, Angelo Avogaro

**Affiliations:** 1 Department of Medicine and Surgery, Università degli Studi di Milano Bicocca, Milan, Italy; 2 Department of Medicine and Rehabilitation, Policlinico di Monza, Via Modigliani 10, 20900 Monza, Italy; 3 Unit of Metabolic Disease, University Hospital of Padua, Padua, Italy; 4 Department of Clinical and Experimental Medicine, University of Pisa, Pisa, Italy; 5 CISUP, Center for Instrument Sharing, University of Pisa, 56124 Pisa, Italy; 6 Department of Medical and Surgical Sciences, University Magna Graecia of Catanzaro, 88100 Catanzaro, Italy; 7 Department of Medicine and Translational Surgery, Università Cattolica del Sacro Cuore, Rome, Italy; 8 Digestive Disease Center, Fondazione Policlinico Universitario Agostino Gemelli IRCCS, Rome, Italy; 9 Department of Medicine and Aging Sciences, Center for Advanced Studies and Technology (CAST, Ex CeSIMet) G. d’Annunzio University Chieti-Pescara, Chieti, Italy; 10 Endocrinology and Metabolism Unit, Pescara Health Service, Pescara, Italy; 11 Sant’Anna School of Advanced Studies, Pisa, Italy; 12 Department of Precision and Regenerative Medicine and Ionian Area, Section of Internal Medicine, Endocrinology, Andrology and Metabolic Diseases, University of Bari Aldo Moro, 70124 Bari, Italy; 13 Department of Clinical and Experimental Medicine, University of Catania, Catania, Italy; 14 Department of Surgical, Medical, Molecular and Critical Area Pathology, University of Pisa, Pisa, Italy

**Keywords:** Incretin, GLP1, GIP, Tirzepatide, Diabetes

## Abstract

Type 2 diabetes represents a growing challenge for global public health. Its prevalence is increasing worldwide, and, like obesity, it affects progressively younger populations compared to the past, with potentially greater impact on chronic complications. Dual glucagon like peptide 1 (GLP1) and glucose-dependent insulinotropic peptide (GIP) receptor agonists are among the new pharmacological strategies recently developed to address this challenge. Tirzepatide, characterized by its ability to selectively bind and activate receptors for the intestinal hormones GIP and GLP-1, has been tested in numerous clinical studies and is already currently authorized in several countries for the treatment of type 2 diabetes and obesity. In this context, the aim of the present document is to summarize, in the form of a narrative literature review, the currently available data on the main mechanisms of action of GIP/GLP-1 co-agonists and the clinical effects of tirzepatide evaluated in various clinical trials.

## Introduction

Type 2 diabetes mellitus (T2DM) and obesity pose a growing challenge to public health, with millions of people affected by these pathological conditions and socio-economic costs steadily increasing worldwide. In Italy, one in twenty adults aged 18–69 years has been diagnosed with diabetes, and four in ten adults are overweight [1]. The prevalence of these conditions rises with age and is higher in men than women and in individuals with lower incomes and education.

In parallel, the landscape of pharmacological therapies for T2DM and (to a lesser extent) obesity has expanded in recent decades with new classes of molecules coming into the market. These novel therapies have often exceeded expectations by demonstrating beneficial effects that extend to complications that were not the primary focus of their development. Among these new therapeutic options, tirzepatide has gathered significant interest in the scientific and medical community as the first member of a new class of drugs characterized by their ability to selectively bind and activate the receptors for the intestinal hormones GIP and GLP-1. Tirzepatide has received approval for use in patients with T2D following several phase 3 studies testing and demonstrating its marked anti-hyperglycemic efficacy and positive actions on multiple cardiovascular risk factors, associated with an excellent safety profile [2, 3]. Approval for the treatment of obesity, irrespective of the presence of diabetes, has also been grated [4]. Additionally, further investigations are underway to explore potential additional uses of tirzepatide in the clinical management of other cardiometabolic conditions associated with obesity, including heart failure and non-alcoholic (or metabolic-dysfunction associated) steatohepatitis (NASH/MASH).

In this narrative literature review, we explore the pharmacological properties of tirzepatide and critically summarize and comment on the results of the clinical studies conducted thus far, as well as discuss the potential implications for the treatment of obesity, T2DM, and associated cardiometabolic complications.

### New evidence on the actions of GLP-1

GLP-1 is secreted by L cells located in the distal ileum and colon in response to nutrient ingestion. The interaction of GLP-1 with its receptor leads to an increase in intracellular cAMP levels and activation of numerous cellular processes that vary depending on the organ or system involved [5]. Among the most well-known and studied effects of GLP-1 is its impact on the pancreatic islet, where the activation of GLP-1-associated signaling pathways results in increased insulin secretion and reduced glucagon production. This response promotes proper nutrient metabolism by increasing glucose utilization in insulin-dependent tissues such as muscle and adipose tissue, reducing endogenous glucose production, and enhancing glycogen synthesis.

GLP-1 is also responsible for a multitude of extra-pancreatic effects that made its pharmacological development even more interesting. This has led to the creation of GLP-1 agonists and analogues capable of effectively activating all receptor and cellular systems compatible with GLP-1. These agonists/analogues of GLP-1 slow down gastric emptying, modulate calorie intake by increasing the sense of satiety, modulate cardiovascular activity, and regulate natriuresis at the renal level [6]. Recently, several pieces of evidence have expanded our understanding of the effects of GLP-1, demonstrating innovative actions even in well-known targets such as the pancreatic islet. In particular, GLP-1 analogues have shown direct effects on the plasticity of the pancreatic islet in both normal glucose tolerance and throughout the whole spectrum of alterations leading to overt diabetes [7]. Evidence indicates that the pancreatic islet is dynamic, plastic, and characterized by processes of trans-differentiation and de-differentiation in a high percentage of T2DM subjects. GLP-1, in addition to its known effects on stimulating beta-cell proliferation and inhibiting apoptosis, is involved in the modulation of trans-differentiation and de-differentiation processes [7].

Through the analysis of numerous in vivo and in vitro studies aimed at determining the mechanisms by which GLP-1 enhances insulin secretion in individuals with normal glucose tolerance or T2DM, two main intracellular mechanisms underlying the incretin effect have emerged. The first mechanism involves the enhancement of the reloading of the insulin pool available for immediate release, occurring only in the presence of incretin and at a certain glucose threshold. The second mechanism is characterized by the modulation of intracellular calcium, which seems to function as a trigger for rapid insulin exocytosis and complements the amplification phenomena [8].

The functional and structural modulation of the pancreatic islet mediated by GLP-1 also has an impact on liver pathophysiology. The complex interaction of factors such as insulin resistance, glucotoxicity and lipotoxicity contribute to the pathogenesis and coexistence of diabetes and metabolic-dysfunction associated steatotic liver disease (MASLD) in a reciprocal process of exacerbating the underlying conditions [9]. Diabetes promotes the progression of MASLD to steatohepatitis, cirrhosis, and hepatocellular carcinoma. GLP-1 agonists/analogues show beneficial effects on most of the multiple alterations underlying MASLD and cirrhosis progression [10]. On the other hand, it is still not entirely clear whether the hepatic effects of GLP-1 are direct or indirect, as recent studies have not clearly demonstrated the presence of GLP-1 receptors in human hepatocytes. A recent in vivo study in healthy subjects showed that acute intravenous infusion of GLP-1 during a pancreatic clamp, in which insulin and glucagon levels were maintained at basal levels, caused a reduction in hepatic glucose production, suggesting a potential direct effect on gluconeogenesis [11]. Another study showed that acute administration of exenatide was associated with increased hepatic glucose uptake during an oral glucose tolerance test (OGTT) and a reduction in endogenous glucose production, reinforcing the hypothesis of a direct action of GLP-1 on the liver [12].

The gastrointestinal system is highly integrated with the central nervous system (CNS). Enteroendocrine cells (EECs), the microbiota, and metabolites produced by the microbiota constitute a complex signaling system [13] that involves the enteric plexus, CNS afferents and efferents, and the modulation of numerous brain nuclei like the nucleus tractus solitarius, hypothalamus, thalamus, and many others directly involved in the control of glucose and energy homeostasis [14]. GLP-1 has been shown to effectively contribute to the functional integration between the CNS and peripheral metabolism; modulation of calorie intake, satiety, and energy balance are just a few of the most evident effects caused by these actions [15]. In recent years, the effects of GLP-1 agonists/analogues on the CNS have gained relevance due to emerging evidence supporting the neuroprotective effects of these molecules. Diabetes is associated with an increased risk of developing dementia and neurodegenerative diseases, which is inversely related to the degree of glucose tolerance and directly to the disease duration [16]. On the other hand, strict glucose control does not limit or improve the damage caused by diabetes and its comorbidities to the CNS [17, 18] as the impact of insulin resistance, obesity, and diabetes on the CNS begins very early in the natural history of the disease [19]. CNS plasticity and cerebral glucose uptake are directly modulated by GLP-1 receptor activation [20, 21]. The potential clinical implications of these observations have been confirmed by a recent multicenter trial involving over 4000 patients, in which dulaglutide resulted in approximately a 14% reduction in the risk of cognitive decline after 5 years of therapy [22]. The neuroprotective effect of GLP-1 is also evident in intervention studies on neurodegenerative diseases such as Alzheimer’s and Parkinson’s. Individuals with diabetes have an increased risk of developing neurodegenerative diseases, and the combination of cerebral and peripheral insulin resistance constitutes one of the main mechanisms underlying the initiation and progression of neurodegenerative diseases [23]. In a trial conducted on a small number of subjects with Alzheimer’s disease, treatment with liraglutide was able to prevent the progressive decline in cerebral glucose uptake that characterizes patients with the disease [24]. Similarly, patients with Parkinson’s disease showed an improvement in motor parameters according to the criteria of the Movement Disorders Society Unified Parkinson’s Disease Rating Scale (MDS-UPDRS) after treatment with exenatide [25, 26]. Therefore, GLP-1 agonists/analogues represent a continuously evolving class of drugs that can modulate mechanisms aimed at achieving increasingly ambitious glycemic and body weight goals. Additionally, the pleiotropic actions of GLP-1 expand the therapeutic potential of GLP-1 agonists/analogues through combination with other drug classes, suggesting new scenarios in terms of enhancing cardio-renal protection and expanding clinical indications.

### Biological effects of GIP

**Fig. 1:**
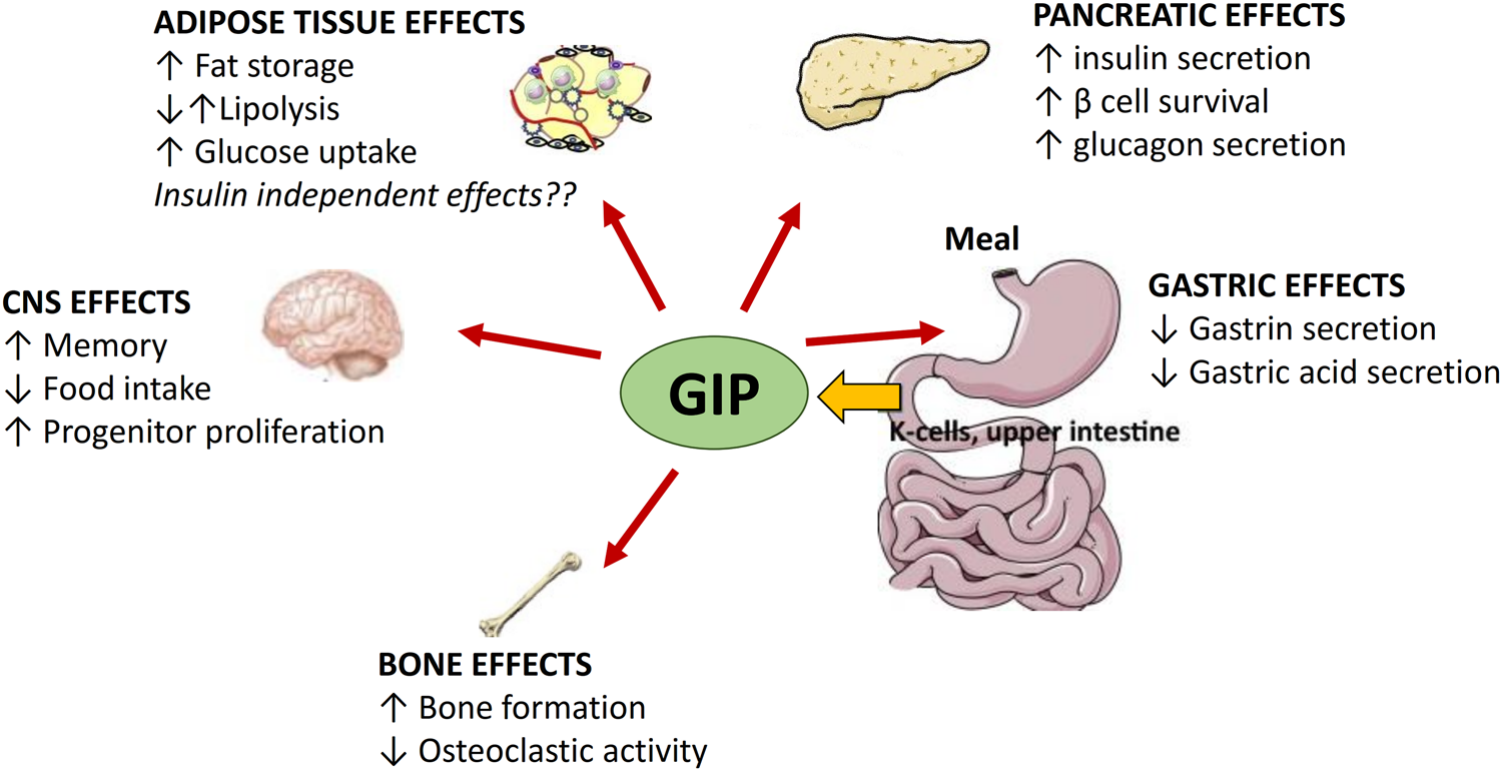
Gastro-intestinal and extra gastro-intestinal effects of glucose-dependent insulinotropic peptide

The Gastric Inhibitory Polypeptide (GIP), isolated for the first time from porcine small intestine in 1971 [27], was named as GIP on the basis of its ability to inhibit gastric hydrochloric acid secretion [28]. It is a 42-amino acid peptide secreted by the K-cells, present in high density in the duodenum and upper jejunum, following oral ingestion of nutrients such as glucose, amino acids, and long-chain fatty acids (Fig. 1). Incretin function of GIP has emerged some years after its identification [29]. Several pieces of evidence have demonstrated that GIP administration results in an improved glucose-dependent insulin secretion [30, 31]. Similarly to GLP-1, GIP exerts several beneficial effects on β cells. Indeed, GIP administration reduces β cell apoptosis and enhances β cell mass in animal models [32]. Additionally, GIP modulates α cells function in a glucose-dependent manner. In hypoglycemic and normoglycemic conditions, GIP administration stimulates glucagon secretion in healthy subjects. On the other hand, during a hyperglycemic clamp, GIP has no effect on glucagon secretion [30, 31]. Interestingly, several alterations in intra-pancreatic actions of GIP have been found in subjects affected by T2DM. Incretin action of GIP is dramatically reduced in the presence of diabetes, as demonstrated by the evidence that glucose-dependent insulin secretion is not potentiated by GIP administration in subjects with diabetes [33, 34]. On the contrary, the positive effect of GIP on glucagon secretion is preserved [34]. The reduced incretin effect of GIP in diabetic subjects may be due to the down-regulation of GIP receptor expression, which has been found in pancreatic islets of diabetic Zucker rats and reverted following correction of hyperglycemia [35].

Notably, GIP exerts several metabolic actions on extra-pancreatic tissues including adipose tissue, brain and bone (Fig. 1). Studies exploring the effects of GIP on adipose tissue have provided controversial results. Deletion of GIP receptor has been found to counteract high-fat diet induced weight gain in mice, suggesting a causal role of GIP in development of obesity [36]. Moreover, GIP administration reduces lipolysis-related genes expression in adipose tissue of overweight subjects and consequently decreases circulating free fatty acid levels [37]. On the other hand, Timper et al. [38] described an augmented lipolysis in differentiated human preadipocyte-derived adipocytes upon GIP stimulation. This divergent evidence may be due to the different experimental conditions of the studies; moreover it is still unclear whether GIP has direct, insulin independent, metabolic effects on adipose tissue. GIP receptor has been found to be expressed in the central nervous system. In particular, mature neurons and progenitor cells in the adult rat hippocampus express GIP receptors, and treatment with GIP is able to promote neuronal cell proliferation [39]. Additionally, GIP contributes to the regulation of hunger sensation. Indeed, acute activation of GIP signaling in hypothalamic cells results in a decreased food intake in rodents [40]. Furthermore, several preclinical studies have demonstrated the beneficial effects of GIP on bone mass. GIP has been found to directly stimulate bone formation by osteoblastic cells and inhibit osteoclastic activity [41, 42]. The protective effects of GIP on the bone have been also observed in humans with and without diabetes [43, 44].

### Effects of tirzepatide on body weight

GLP-1 receptor agonists, initially developed and marketed to exploit their relevant hypoglycemic effects, have shown positive but heterogeneous effects on body weight control. These effects depend on the type, route of administration, dose, and duration of action of each specific GLP-1 receptor agonist, as well as individual characteristics such as baseline body weight and degree of glucose tolerance. In the SCALE study, liraglutide, administered daily at a dose of 0.6–3.0 mg, was associated with a body weight loss 5.6 kg greater than placebo [45].

The efficacy of liraglutide on weight management was subsequently surpassed by semaglutide, which, when administered weekly at a dose of 2.4 mg in obese patients without diabetes, resulted in a weight reduction of 12.4% compared to placebo in the STEP 1 study [46], and a reduction of 9.4% compared to liraglutide 3.0 mg in the STEP-8 study [47]. With the development of the first GIP/GLP-1 co-agonist, tirzepatide, there has been an exponential increase in the effectiveness of incretin-mimetic pharmacological therapy for weight control in obese patients with and without diabetes. In subjects with T2DM, weekly administration of tirzepatide at doses of 5 mg, 10 mg, or 15 mg was associated with greater weight loss compared to placebo (− 6.3 kg, − 8.4 kg, and − 9.4 kg, respectively), and 1.7 kg, 4.8 kg, and 7.2 kg greater weight loss compared to selective GLP-1 receptor agonists, demonstrating a dose-dependent effect sustained for up to 2 years [48].

In the SURPASS-2 study, tirzepatide 15 mg resulted in a faster and greater decline in body weight (− 5.5 kg) compared to semaglutide 1.0 mg, more than doubling the proportion of patients achieving at least 10% weight loss (57% vs 24%) [3]. Greater weight loss efficacy of tirzepatide 10 mg (− 3.2 kg) and 15 mg (− 5.2 kg) compared to semaglutide was also observed in an indirect comparison with data from the SUSTAIN FORTE study, where semaglutide was administered at the maximum hypoglycemic dose of 2.0 mg [49].

The effects of tirzepatide on body weight control have also been confirmed in non-diabetic individuals in the SURMOUNT-1 study, where treatment with tirzepatide 10–15 mg was associated with an average weight loss of approximately 20% at 72 weeks, exceeding 25% in one third of the patients. It is interesting to note that the positive effect of tirzepatide on body weight is associated with a significant improvement in quality of life and physical performance [50] and is independent of sex [51], baseline body mass index (BMI) [52] and potential side effects, predominantly gastrointestinal [53]. Furthermore, the weight loss induced by tirzepatide appears to be primarily due to a greater reduction in fat mass compared to metabolically active lean mass, resulting in a favorable redistribution of abdominal ectopic adipose tissue and a marked reduction in waist circumference and intrahepatic fat [54]. The exact mechanisms underlying tirzepatide-induced weight loss remain to be clarified. The simultaneous activation of GIP and GLP-1 receptors may have central synergistic effects on appetite control, greater than selective GLP-1 receptor agonists [55], although preliminary studies do not support this hypothesis [56]. Alternative and non-mutually exclusive mechanisms may involve favorable effects on basal energy expenditure [57] or on the choice of less energy-rich foods consumed in free-living conditions [58], although these latter hypotheses remain to be validated in clinical studies.

### Glucose control

The SURPASS studies enrolled populations that were similar in terms of baseline HbA1c levels, which ranged from 7.9 to 8.5% [2, 3, 59–61] and found a statistically significant reduction in HbA1c already at the 5 mg dose, demonstrating a greater efficacy of tirzepatide compared to placebo or active comparator [2, 3, 59–61]. Interestingly, the reduction in HbA1c was already evident after the first 4 weeks of treatment and remained statistically significant up to the end of the 52-week observation period, supporting the evidence of rapid efficacy of the drug and great durability, which was independent from weight loss.

In terms of achievement of HbA1c target, 81% and 97% of patients treated with tirzepatide reached an HbA1c less than 7%, while 71% and 95% of patients achieved a HbA1c of less than 6.5%. In addition, 23% and 61% of patients achieved HbA1c values below 5.7%, with no documented hypoglycemia episodes in SURPASS 1 (vs. add on to other therapeutic schemes). It is interesting to note that the efficacy of Tirzepatide evaluated on a 7-point daily glucose profile demonstrated better control at all points of the glycemic profile compared to semaglutide 1 mg [3]; on the other hand, the comparison with insulin degludec showed that although insulin, titrated during the study, obtained a better result on fasting blood glucose, treatment with tirzepatide 10 and 15 mg demonstrated better control on all other points of glucose profile [60].

In addition to the improvement in HbA1c and glucose profile, tirzepatide has also demonstrated to improve insulin resistance, as assessed by HOMA-IR. This effect was independent from the dose of tirzepatide used and was similar in case of placebo [59] or treatment with active comparator semaglutide 1 mg [3].

In order to evaluate the mechanisms of action of tirzipatide, in a recent study [62] subjects with T2DM performed a deep metabolic evaluation with measures of beta cell function and insulin sensitivity by hyperglycemic clamp, mixed meal test and euglycemic clamp before and 28 weeks after treatment with tirzepatide 15 mg or semaglutide 1 mg. This study confirmed that tirzepatide induces a great improvement in insulin sensitivity compared to semaglutide, as already observed with HOMA-IR. In addition, a significant improvement in the first and second phase of insulin secretion was observed with tirzepatide treatment, as well as an improvement in beta cell function, estimated with beta-cell glucose sensitivity. Compared with semaglitude 1 mg, treatment with tirzepatide demonstrated reduced glucagon secretion in response to mixed meal, suggesting an additive effect compared to GPL1 RA on glucagon suppression.

The efficacy of GIP/GLP1 receptor co-agonists on glucose control is surprising, and allows to reach glucose levels similar to diabetes remission in a greater percentage of patients compared to other treatments. The mechanisms of action of tirzepatide are multiple and need to be further investigated, but the results obtained so far suggest an effect both in improving insulin resistance and in enhancing beta cell function by restoring insulin secretion.

### Prevention of cardiorenal complications and beyond

With the improvement in the management of cardiovascular risk factors, a progressive decline in the annual incidence rate of cardiovascular morbidity and mortality has been observed in both the general population and the diabetic population over the past decades. However, this reduction has progressed in parallel among groups of patients with and without diabetes, and the gap in CVD risk between individuals with and without diabetes is unchanged [63]. At the same time, the incidence of diabetic nephropathy, although reduced compared to 20 years ago, is stabilized in recent years, and the incidence of end-stage renal disease (due to reduced cardiovascular mortality and increased life expectancy) is increasing [64]. Furthermore, the secular trends of causes of mortality in patients with diabetes shows an increase for neurodegenerative causes (e.g., dementia) and liver diseases [65]. This leads to a scenario where new therapeutic strategies for diabetes must inevitably consider not only glycemic and weight control but also the reduction of both vascular and non-vascular complications. In this regard, the GIP and GLP-1 agonist, tirzepatide, currently shows promising data derived from phase 2 and 3 clinical trials.

*Hepatic Steatosis*: Recently, a sub-study of the phase 3 clinical trial SURPASS-3 showed that, among patients receiving metformin and/or SGLT2 inhibitors, the addition of tirzepatide (10 or 15 mg) as compared to insulin degludec was able to significantly reduce hepatic fat content (evaluated by magnetic resonance) after 52 weeks of treatment. The absolute difference in liver fat content [LFC] was − 4.7% (95% CI from − 6.7 to − 2.7), with a relative decrease in LFC from baseline of − 35.9% and − 28.4% for the two individual dosages (*p* < 0.0005), and a significant reduction of − 18.6% even at the 5 mg dosage. This reduction in LFC was largely explained by weight loss and improved glycemic control induced by tirzepatide (50%), although it is possible to hypothesize that the remaining effect may be related to improvements in lipotoxicity, inflammation, and mitochondrial function caused by tirzepatide [54]. These data will need biopsy evaluation to assess the benefits of tirzepatide on the histological features of NAFLD/NASH or fibrosis, which are currently being collected in an ongoing clinical trial (SYNERGY-NASH-NCT04166773).

*Diabetic Kidney Disease*: The effect of tirzepatide on the progression of renal damage has been evaluated so far in the post-hoc analysis of the SURPASS-4 study (phase 3, open-label), which randomized patients receiving metformin, sulfonylurea, or SGLT2 inhibitors to tirzepatide (5 mg, 10 mg, or 15 mg weekly) versus insulin glargine (100 U/ml) for 104 weeks. After a median of 85 weeks, the decline in estimated glomerular filtration rate (eGFR) was 2.2 ml/min/1.73m^2^/year (95% CI 1.6 to 2.8) in favor of tirzepatide, accompanied by a difference in the urinary albumin/creatinine ratio (uACR) of − 31.9% (95% CI from − 37.7 to − 25.7%), also in favor of tirzepatide. The composite renal outcome (ESRD, eGFR decline > 40%, renal death, new onset of macroalbuminuria) strongly favored tirzepatide (HR 0.58, 95% CI from 0.43 to 0.80, *p* = 0.0008); such results were primarily driven by the reduced incidence of macroalbuminuria. The relatively small numbers (n = 1989) and the short duration of the study require caution, but the results appear highly promising while awaiting the results of ongoing studies (SURPASS-CVOT) [66].

*Cardiovascular Safety*: The cardiovascular safety of tirzepatide has been evaluated and confirmed to date through a pre-specified meta-analysis of phase 2 and 3 randomized trials comparing tirzepatide with other placebo, insulin glargine, degludec, semaglutide, dulaglutide for a minimum of 26 weeks (including the GPGB, SURPASS 1,2,3,4,5, and J-mono studies) [67]. The analysis of 4887 participants treated with tirzepatide and 2,328 participants in the control groups identified 142 participants who developed at least one MACE-4p (inclusive of cardiovascular mortality, myocardial infarction, stroke, and hospitalization for unstable angina) and confirmed the cardiovascular safety with an hazard ratio (HR) of 0.80 (95% CI 0.57–1.11) for the comparison between tirzepatide and controls. This finding was consistent for cardiovascular mortality (HR 0.90, 95% CI from 0.50 to 1.61) and all-cause mortality (HR 0.80, 95% CI from 0.51 to 1.25).

However, it is important to exercise caution in interpreting these hazard ratios. On one hand, the confidence intervals only allow for conclusions regarding non-inferiority, and on the other hand, the average duration of observation was only 1 year. It should also be noted that, from a methodological perspective, the model used (Cox with stratification by cardiovascular risk class of the trials, i.e., SURPASS-4: high risk, and all others: low risk) requires various assumptions, including the homogeneity of the relative effect of tirzepatide treatment on MACE regardless of the baseline cardiovascular risk level of the patients. This assumption is desirable but will need to be confirmed in subsequent studies. Therefore, for now, it is better to focus on the conclusions that tirzepatide is safe from a cardiovascular perspective. However, it is only a matter of time before we will have more definitive answers. Indeed, the SURPASS-CVOT study, which compares tirzepatide head-to-head with dulaglutide, will likely provide more comprehensive answers (expected by the end of 2024).

### Perspectives on clinical impact and adherence

The World Health Organization defines adherence as “the extent to which a person’s behavior—taking medication, following a diet, and/or executing lifestyle changes, corresponds with agreed recommendations from a health care provider” [68]. Several factors influence the patient’s level of adherence and can be distinguished as social and economic factors, patient-related factors (health beliefs, health literacy), therapy-related factors (complexity of the treatment, adverse events), presence of comorbidities (neurological/psychiatric or of different origins), and factors related to the healthcare system (doctor-patient relationship, difficulty in obtaining follow-up visits). These barriers account to varying degrees for the reduced level of adherence described for multiple asymptomatic chronic conditions, including diabetes. A meta-analysis has indeed highlighted that only about 50–60% of patients with diabetes adhere to antidiabetic therapy [69], with a significant impact on quality of life and life expectancy. Several real-world studies based on administrative data and pharmacy reports have demonstrated that reduced adherence to antidiabetic therapy is associated not only with poorer glycemic control but also with a significant increase in overall mortality and hospitalizations for all causes [70, 71].

There are several interventions capable of promoting adherence to pharmacological treatment. Education plays a critical role, as demonstrated by various randomized trials [72]. It is essential to verify the patient’s understanding of the prescribed therapeutic regimen, clarify the benefits that can result from the therapy, discuss possible adverse events, and provide useful advice to minimize them, as well as simplify the therapeutic regimen when appropriate [73]. While real-world data on the level of adherence to dual GLP1-RA/GIP agonists are not available to date, some considerations can be drawn from available clinical trials and experiences with other pharmacological classes. In the SURPASS program, gastrointestinal side effects associated with the use of tirzepatide were similar to those observed with semaglutide and GLP1-RA drugs in general, both in terms of frequency and duration [3]. Tirzepatide has also demonstrated superior efficacy compared to any other therapy used in the treatment of T2DM, comparable or superior even to regimes based on the use of multiple pharmacological classes. Since several studies document a decrease in adherence as therapeutic complexity increases, an improvement in therapeutic adherence can be expected with the use of this drug, especially considering the excellent results that patients can achieve in terms of weight reduction. Lastly, as demonstrated by a large online survey, patients with T2DM generally have a positive attitude towards the option of a weekly-administered antidiabetic therapy rather than a daily one [74], especially when it involves a user-friendly device [75]. These data instill optimism about the impact that this drug might have in the treatment of T2DM and its complications in every day clinical practice.

## Conclusions

The currently available evidence on the mechanisms of action and clinical effects of GLP-1/GIP dual agonism supports very optimistic prospects for the future use of tirzepatide. This new molecule, by acting synergistically on combined systems known to be altered in T2DM [33, 34], could indeed expand the therapeutic potential of GLP-1 agonists/analogues, suggesting new scenarios in terms of improving glycemic control, weight management, and hopefully providing protection against chronic micro-macrovascular complications and not only limited to hepatic complications.

The potential of this molecule should be understood within the context of the epidemiological evolution of T2DM, characterized by several key elements, including: 1. The progressive increase in prevalence both globally and nationally [1]. 2. The reduction in the average age of onset of diabetes (and obesity) [76] 3. The greater impact on morbidity and mortality of diabetes in patients who develop the condition at a younger age compared to those who develop it later [77]. Therefore, while efforts undoubtedly need to be intensified towards preventing the onset of the disease and making lifestyle modifications (a fundamental aspect of the therapy for every patient with T2DM), it is clear, as indicated by the most recent guidelines [78], that the pharmacological approach should focus on timely treatment that leaves no room for therapeutic inertia and employs medications that are as effective as possible in both metabolic control and the prevention of chronic complications.

In this context, the data currently available on tirzepatide, showing its efficacy in glycemic control to the extent that a considerable percentage of patients achieve glucose levels indicative of diabetes remission [3, 59], as well as its high effectiveness in weight management, are promising regarding its future use in clinical practice. These clear effects (observed through comparison with various comparators, including current weekly GLP1-RAs on the market) are complemented by interesting data on cardiovascular safety and efficacy (from post-hoc studies) regarding the incidence and progression of nephropathy and hepatic steatosis. It is still too early to draw definitive conclusions, but the numerous ongoing clinical trials, including the cardiovascular safety trial versus an active comparator (dulaglutide), the first of its kind, will provide us shortly with important confirmations.
